# Pulmonary Complications of Cancer Therapy: Clinical Presentations, Imaging Patterns, and Management Strategies

**DOI:** 10.3390/medicina62030578

**Published:** 2026-03-19

**Authors:** Bilal Zafar, Tasmea Haque, Miranda Tan, Ritika Singh, Lara Bashoura, Ajay Sheshadri, Maria Azhar, Saadia A. Faiz

**Affiliations:** 1 Division of Pulmonary, Critical Care & Sleep Medicine, McGovern Medical School at UTHealth, Houston, TX 77030, USA; bilal.zafar@uth.tmc.edu (B.Z.); tasmea.k.haque@uth.tmc.edu (T.H.); 2 Division of Sleep Medicine, Department of Psychiatry, Stanford University School of Medicine, Palo Alto, CA 94305, USA; tanm@stanford.edu; 3 Department of Internal Medicine, Baylor College of Medicine, Houston, TX 77030, USA; ritika.rs.singh@gmail.com; 4 Department of Pulmonary Medicine, The University of Texas MD Anderson Cancer Center, Houston, TX 77030, USA; lbashoura@mdanderson.org (L.B.); asheshadri@mdanderson.org (A.S.); 5 Bayhealth Medical Group (Kent Campus), Dover, DE 19901, USA; mariaazhar73@gmail.com

**Keywords:** pneumonitis, immunotherapy, targeted, therapies, radiation-induced lung injury, pulmonary vascular disease

## Abstract

*Background and objectives:* Therapeutic agents for cancer can cause unique pulmonary toxicities and mimic other conditions. The advent of new targeted molecular and immune therapies has changed the landscape of cancer treatment. These adverse events pose diagnostic and therapeutic challenges. This review aims to summarize the clinical presentations, radiographic patterns, and management strategies for noninfectious pulmonary complications associated with cancer therapies. *Materials and methods:* A literature review was conducted focusing on drug-induced lung injury (DILI), radiation-induced lung injury (RILI), pleural disease, pulmonary vascular complications, and other inflammatory conditions in patients with cancer. The data sources included clinical trials, guideline recommendations, observational studies, and expert consensus addressing incidence, pathophysiology, imaging findings, and treatment approaches. *Results:* Noninfectious pulmonary sequelae of anti-neoplastic therapies encompass a broad spectrum of etiologies. DILI occurs in up to 30% with variable onset and severity. The patterns can be diverse but include interstitial pneumonitis, organizing pneumonia, and diffuse alveolar damage. RILI is common and influenced by the radiation dose, volume, and concurrent therapies, and it may have both acute and chronic clinical and radiographic presentations. Pleural disease may arise from radiation and other agents, and the determination of etiology can impact management. Pulmonary vascular disease arises from many different etiologies, including therapies such as tyrosine kinase inhibitors and proteosome inhibitors, thromboembolic disease, as well as rare processes, including pulmonary veno-occlusive disease. Other conditions such as transfusion-related lung injury, cryptogenic organizing pneumonia, and interstitial lung abnormalities can also further complicate the diagnosis. *Conclusions:* Noninfectious pulmonary complications related to cancer therapies are diverse and often indistinguishable from infectious or malignant processes. The integration of clinical history, imaging, and selective invasive testing are needed for a timely diagnosis. Management typically involves withdrawal of the offending agent and corticosteroids, with immunosuppressive therapy reserved for severe or refractory cases. The awareness of these entities and early recognition are critical to optimizing outcomes.

## 1. Introduction

Pulmonary symptoms in patients with cancer can signal infection, inflammation, or malignancy. Therapeutic anti-neoplastic agents potentially cause significant pulmonary toxicities, and new therapies have changed the landscape of cancer treatment. Often, noninfectious pulmonary manifestations may create diagnostic challenges. Additional malignant processes, whether synchronous or metastatic, can also emerge. Different grading symptoms exist to define adverse events (Table 1), and the stratification of symptoms and pneumonitis severity helps to guide management interventions [1,2]. Given the advent of new targeted molecular and immune therapies, a timely review on potential adverse pulmonary sequelae is needed, thus this narrative review focuses on noninfectious clinical entities with an emphasis on clinical presentations, radiographic findings, and treatment strategies.

## 2. Methods

We performed a literature review using the Texas Medical Center Library database and PubMed Central for articles published between 1 January 2014 and 1 January 2025. We used medical subject heading terms to search titles, abstracts, and diagnoses. We reviewed textbook chapters, the literature reviews, practice guidelines, randomized controlled trials, and observational studies. While the mechanisms of lung injury are not fully defined for many agents, they vary from cytotoxic agents to targeted therapies and immune-mediated drugs. Online databases such as the Food and Drug Administration drug label and Pneumotox Online (https://pneumotox.com/drug/index/ accessed on 1 January 2025) are also helpful to identify agents and adverse events. When available, recommendations from society guidelines were prioritized, and information regarding study design (prospective, randomized, or observational) and/or expert consensus statements were shared. 


**Items**

**Specification**
Date of search1 January 2014 to 1 January 2025Databases and other sources searchedPubMed CentralSearch terms used
Cancer therapy, chemotherapy, immunotherapy, targeted therapy, radiation therapy with pulmonary toxicity, lung injury, pneumonitis, interstitial lung disease, respiratory complications.

Immune checkpoint inhibitors, tyrosine kinase inhibitors, biologic therapy with pneumonitis and pulmonary adverse effects.Radiation pneumonitis, radiation fibrosis, thoracic radiotherapy with lung injury.Drug-induced lung disease, treatment-related pneumonitis, organizing pneumonia, diffuse alveolar damage.Imaging patterns (ground-glass opacities, non-specific interstitial pneumonitis, organizing pneumonia, diffuse alveolar damage) associated with cancer therapy.Free-text search terms were used for uncommon entities (e.g., “radiation recall pneumonitis,” “drug-induced organizing pneumonia”).

Reference lists of these papers were used to identify relevant case series, large cohort series, and meta-analyses.
Timeframe2010 to 2025Inclusion and exclusion criteria
Textbook chapters, literature reviews, practice guidelines, randomized controlled trials, and retrospective reviews were included.

Only articles written in English were included.
Selection processIndependent. All selected articles were reviewed by all authors.

## 3. Drug-Induced Lung Injury

Drug-induced lung injury (DILI) encompasses a highly heterogeneous spectrum of adverse drug reactions and has been reported with numerous pharmacologic classes [3]. Cytotoxic agents are associated with both acute and chronic lung injury, with multiple drug classes implicated (Table 2) [4,5]. Molecular targeted therapies have transformed personalized cancer care; however, many of these agents are also associated with pulmonary toxicity (Table 2) [6,7,8,9]. The focus will be to provide an overview of DILI presentation and workup, to briefly mention those cytotoxic agents commonly associated with pulmonary toxicities, and to highlight immune-mediated and new targeted therapies. Given the wide range of implicated therapies, this section focuses on agents most often associated with lung injury, acknowledging that some may not be included.

The incidence of DILI varies widely, ranging from 10% to 30%, and it is influenced by the offending agent and patient-specific factors, including comorbid conditions, prior cancer therapies and concurrent medications [10]. DILI presents a significant diagnostic challenge, as no clinical, laboratory, radiographic, or histologic features are pathognomonic. Clinical outcomes are similarly heterogeneous, with prognosis determined by the causative drug, disease context, and host factors, including the underlying malignancy.

Clinical manifestations can vary, and respiratory symptoms range from mild (cough, dyspnea, hemoptysis, and pleurisy) to those with systemic signs including fever, fatigue, or malaise. The symptoms may develop acutely or insidiously over the course of weeks to months after the initiation of therapy [11]. Although the temporal exposure to the drug is helpful in elucidating the diagnosis, DILI can also be idiosyncratic, without a clear relationship to time or dose. For example, bleomycin and nitrosoureas can cause delayed fibrosis, while immune checkpoint inhibitors (ICIs) have been associated with delayed pneumonitis [11,12].

**Figure 1:**
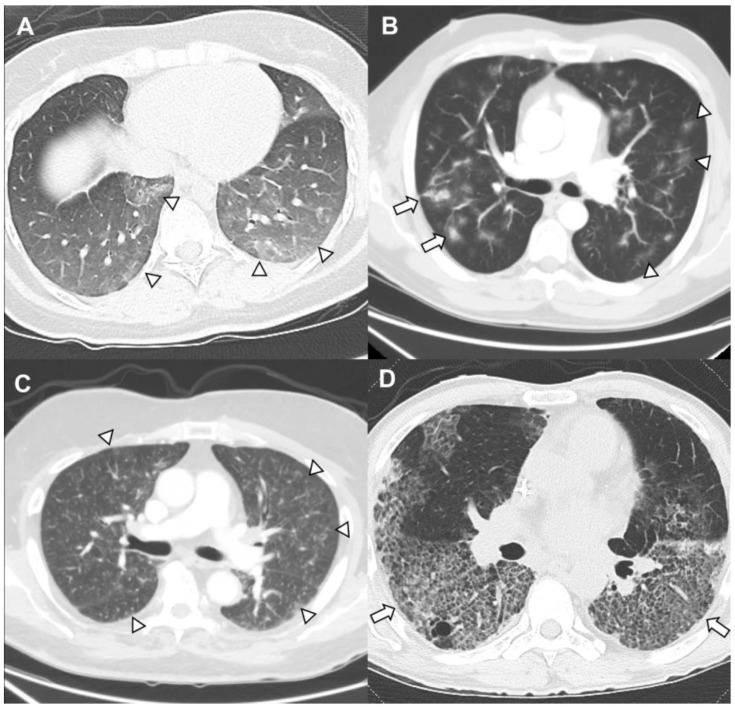
**Drug-induced lung injury (DILI).** (**A**) Bleomycin toxicity developed after infusion in Hodgkin’s lymphoma. Imaging revealed bilateral ground-glass infiltrates (arrowheads) with a lower lobe predominance. (**B**) Immune checkpoint inhibitor pneumonitis in metastatic renal cell carcinoma with organizing pneumonia. Imaging revealed bilateral, multifocal, pan-lobar, irregularly marginated, coalescent airspace opacities with ground-glass halos (arrow heads) with some areas of consolidation (arrows). (**C**) Immune checkpoint inhibitor pneumonitis in thyroid cancer on atezolizumab with bilateral infiltrates. Imaging demonstrated ground-glass and small nodular opacities (arrowheads). (**D**) Interstitial pneumonitis in colorectal cancer from trastuzumab deruxtecan. Imaging showed diffuse ground-glass changes (arrow) with a predominance in the lung bases.

The diagnosis of DILI requires a high index of suspicion and careful review of risk factors, history of drug exposures, clinical presentation, and radiographic abnormalities. The exact pathogenesis remains unclear for many drugs, but DILI likely involves direct injury to the alveolar epithelial and endothelial cells, leading to an inflammatory cascade and increase in capillary permeability [3]. Another common mechanism is the production of reactive oxygen metabolites and the subsequent redox reactions with fatty acid oxidation, thereby causing membrane instability and lung injury. These pathophysiological events yield a wide array of clinical presentations, including interstitial pneumonitis/fibrosis, hypersensitivity syndrome, and noncardiogenic pulmonary edema (capillary leak syndrome). Diffuse alveolar hemorrhage, eosinophilic pneumonia, organizing pneumonia, bronchospasm, and acute lung injury (diffuse alveolar damage) may also occur. Radiographic patterns may be non-specific and can vary from reticular thickening to diffuse ground-glass infiltrates to fibrosis and/or nodules (Figure 1). Other manifestations can include sarcoid-like granulomatosis, eosinophilic pneumonia, organizing pneumonia, non-specific interstitial pneumonitis, and acute respiratory distress syndrome (ARDS) [3,6,8].

**Figure 2:**
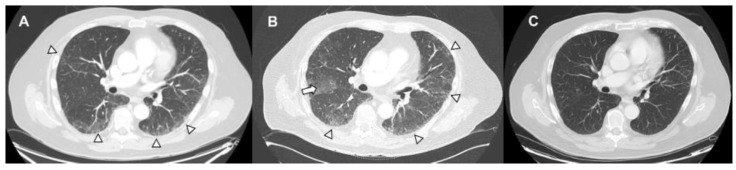
**Pneumonitis related to gemcitabine.** Gemcitabine toxicity in squamous cell carcinoma of the prostate. One month after therapy (**A**), imaging revealed new patchy ground-glass opacities (arrowheads noted in the lung bases). Four months later (**B**), imaging revealed interval increase in subpleural fibrosis (arrowheads) and increased ground-glass opacities (arrows) in both lungs. Imaging 11 months after steroids (**C**) demonstrated resolution of infiltrates.

The diagnosis of DILI is often a diagnosis of exclusion, but the timing of drug exposure, clinical and imaging pattern, concomitant exposures, or disease processes can be helpful. DILI may mimic other respiratory illnesses, such as pneumonia, or other inflammatory processes and metastases, so a respiratory viral panel, sputum, and blood cultures should be obtained. Bronchoscopy is another important diagnostic intervention, for a bronchoalveolar lavage can assess alternative diagnoses, including infection and malignancy [3]. In select cases, transbronchial biopsy may be considered if clinically feasible to evaluate for progressive malignancy, fungal infections, or steroid responsive interstitial lung disease (ILD) [13]. Additional objective assessment with pulmonary function tests may reveal reduction in diffusion capacity and restrictive physiology. The severity grading of DILI (Table 1) ranges from mild to fatal [2]. Withdrawal of the offending drug remains the cornerstone of treatment in suspected DILI, and, in select cases, rechallenging with therapy can be considered but is variable based on the agent. While the role of glucocorticoids in mild DILI has not been established through randomized trials (Figure 2), limited observational data indicate that they may confer benefit [14]. In patients with progressive or severe manifestations, other disease-modifying agents may also be indicated.

### 3.1. Cytotoxic Agents

Chemotherapy is an integral part of cancer treatment but has potential for pulmonary toxicity. The actual incidence of DILI may be unclear due to limited prospective studies, different definitions of DILI, and a lack of confirmation or clinical consensus. As with other DILIs, management includes drug cessation, supportive measures and empiric steroid therapy.

- Bleomycin. Bleomycin has direct cytotoxic effect and induces tumor cell death by inhibition of angiogenesis and generation of free oxygen radicals. Lung and skin toxicities are common due to a lack of the bleomycin-inactivating enzyme, bleomycin hydrolase [15]. Bleomycin-induced pneumonitis can occur in 10% of patients and progress into pulmonary fibrosis, and mortality in severe cases is 60% [16,17]. Risk factors include advanced age, renal function impairment, and thoracic radiation [18].
- Gemcitabine. Gemcitabine-associated pulmonary toxicities include dyspnea (8–10%), interstitial pneumonitis (1.1–3.9%) with patterns of non-specific interstitial pneumonia, and/or hypersensitivity pneumonitis, pulmonary eosinophilia, radiation recall, and ARDS [15,19,20]. In a large Japanese study of 25,924 patients, ILD was observed in 1.7% of those on gemcitabine-based chemotherapy [20]. Toxicity is thought to be a cytokine-mediated inflammatory reaction of the alveolar capillary wall, and a recent pharmacovigilance analysis has identified CD40 as a key pathogenic target [21,22].
- Methotrexate. Methotrexate is an anti-metabolite agent that interferes with folic acid metabolism, and the incidence of methotrexate-associated pneumonitis has been reported to range between 0.3% to 11% [23]. The mechanism is thought to be an idiosyncratic hypersensitivity reaction independent of drug dose.
- Alkylating agents. Alkylating agents are well-recognized for their interstitial pneumonitis and potential for pulmonary fibrosis, and these include cyclophosphamide, busulfan, and nitrosoureas (carmustine, BCNU; lomustine, CCNU) [24,25]. “Busulfan lung,” characterized by insidious onset of dyspnea, dry cough, and restrictive ventilatory impairment occurring months to years after exposure. Nitrosoureas, particularly carmustine, have been associated with dose-dependent pulmonary toxicity with increased risk when cumulative doses exceed 1000–1500 mg/m^2^ or with prior thoracic radiation and/or concurrent use of other pneumotoxic agents [24]. Furthermore, alkylating agents are often part of the conditioning regimens used prior to hematopoietic stem cell transplantation, and these agents can directly injure pulmonary parenchyma and endothelial cells, leading to early interstitial damage and inflammation [26]. This mechanism is thought to contribute to idiopathic pneumonia syndrome occurring post-transplant. Risk is further compounded by concomitant total body irradiation, older age, pre-existing lung disease, and allogeneic donor source.
- Taxanes. Taxanes act by promoting microtubule assembly and preventing their disassembly, thereby inhibiting mitosis and promoting cell death. The incidence of taxane-induced interstitial pneumonitis is rare and varies by formulation with nab-paclitaxel showing higher rates (13%) compared to conventional paclitaxel (<1%) and docetaxel (rare) [7]. However, in a small prospective study of 40 patients with lung cancer on docetaxel, pulmonary toxicity was reported at 4.6% [27].

### 3.2. Immune Checkpoint Inhibitors

ICIs have changed the landscape of cancer therapy with improved survival outcomes in immunogenic tumors. By enhancing T-cell activation, immune checkpoint blockade impairs tumor survival but can also result in toxicity, manifesting as immune-related adverse events (irAEs). The incidence of irAEs ranges from 2.7% to 10% with programmed cell death protein 1/programmed death-ligand 1 (PD-1/PD-L1) inhibitors and is less common (<1%) with cytotoxic T-lymphocyte-associated protein 4 (CTLA-4) inhibitors. [28]. Rates may increase with combination ICI therapy, and coadministration particularly with dual-checkpoint blockage carries 10% incidence of irAE versus 3% for monotherapy [28]. Interestingly, the occurrence of irAEs can be associated with improved overall survival, but this depends on the specific site of toxicity, severity, and timing [29,30].

Real-world data in lung cancer suggest higher incidence rates than those reported in clinical trials, ranging from 9.5% to 15% [31]. The median time to symptom onset is approximately 34 weeks (range: 1.5–127 weeks), although most cases occur within the first 6 to 8 months of therapy [28,32]. The timing is variable and can range from days to over 2 years, and it can occur at any point during therapy or even after treatment discontinuation [28,33]. In a small study of 386 patients with melanoma treated with ICIs who developed pneumonitis (19/386), unprovoked recurrent pneumonitis (3/19) after treatment occurred in some, and this was characterized by early initial onset, shorter initial steroid course, and more severe presentation that the first episode [34].

Patient-specific risk factors may accelerate or delay the onset of toxicity, and ICI-associated pneumonitis can be fatal [35]. Pre-existing lung disease, including obstructive lung disease, ILD, or interstitial lung abnormality (ILA), is the strongest identified risk factor, and it is associated with increased severity and worse outcomes [31,36,37]. Additional risk factors include lung cancer (compared to melanoma or renal cell cancer), lung cancer histology (squamous > adenocarcinoma), prior thoracic radiation, smoking history, and combination ICI therapy [38]. Radiographic patterns are heterogeneous, with organizing pneumonia being most common pattern, followed by non-specific interstitial pneumonia and diffuse alveolar damage [38,39].

Management is guided by symptom severity and established guidelines by the National Comprehensive Cancer Network (NCCN), American Society of Clinical Oncology (ASCO), European Society for Medical Oncology (ESMO), and Society for Immunotherapy of Cancer (SITC) [28,40,41,42]. There is a substantial overlap in the recommendations for pneumonitis management, with holding ICI with observation in Grade 1, systemic steroids in Grade 2, 3, and 4, and higher doses of systemic steroids and addition of second-line therapies for Grades 3 to 4. In general, steroid taper over 4 to 6 weeks is recommended, for unprovoked recurrent pneumonitis has been reported with early initial onset irAE and shorter initial steroid course [34]. Evidence guiding second-line therapy is limited, with agents such as mycophenolate mofetil, infliximab, intravenous immunoglobulin, and tocilizumab used based on institutional experience and disease severity; larger prospective trials are needed to guide management [14,41,43]. Challenging cases include steroid-unresponsive, steroid-resistant, and steroid-dependent irAEs [41].

Rechallenge following the resolution remains controversial, and retrospective data suggest recurrence rates of 25 to 33% [33,44]. The NCCN guidelines recommend the consideration of rechallenge only after there is a resolution of pneumonia (<grade 1) and after the patient is off steroids. Rechallenge is discouraged after high-grade (3 or 4), recurrent, or steroid-refractory disease.

### 3.3. Antibody–Drug Conjugates

Antibody–drug conjugates (ADC) are rapidly emerging as effective treatment modality for various solid organ tumors and hematologic malignancies, with ILD or pneumonitis being their primary pulmonary toxicity. Given its unique mechanism, adverse effects involve both target-independent uptake by alveolar macrophages and payload-related cytotoxicity [45]. A meta-analysis demonstrated that pneumonitis was the most common cause of death associated with ADCs, and fatal pneumonitis has been reported in 2 to 5% [46,47,48].

The overall incidence ranges from 2.7% to 28.0% depending on the ADC, and pneumonitis has been associated with trastuzumab deruxtecan (T-Dx) (13.6%), mirvetuximab soravtansine (10%), enfortumab vedotin (28%), and brentuximab vedotin (3.2%) [46,48,49]. Time to onset is variable dependent on the type of ADC, but for T-Dx-associated pneumonitis, 4 to 7 months has been reported. The median time for enfortumab-related pneumonitis was 13 weeks (range 2.7 to 51 weeks) [50].

Management is grade-dependent and, similar to ICIs, involves drug cessation with observation for Grade 1, the addition of steroid therapy (0.5 mg/kg/day) for grade > 2, and higher dosage for Grade 3 and Grade 4. The data for additional immunosuppressive agents is limited.

Rechallenge with ADCs differs from that with ICIs. Rechallenge with T-DXd after Grade 1 pneumonitis is feasible and appears safe, but rechallenge after Grade > 2 is not recommended [51]. For enfortumab vedotin, drug should be withheld in cases of Grade 2 pneumonitis until recovery to Grade < 1, then treatment may be resumed or dose-reduced [52]. For Grade > 3, it must be permanently discontinued.

### 3.4. Tyrosine Kinase Inhibitors

Tyrosine kinase inhibitors (TKIs) are associated with pneumonitis with varying frequency and severity, and EGFR-TKIs (gefitinib, erlotinib, osimertinib, and dacomitinib) and ALK inhibitors (crizotinib, alectinib, brigatinib, and ceritinib) have all been implicated [14,53]. Among the BCR-ABL TKIs, pneumonitis has been reported with imatinib, but it remains rare [54]. The incidence of EGFR-TKI-associated ILD ranges from 1% to 5% globally, with higher rates reported in East Asian populations. Risk factors include advanced age, smoking history, poor performance status, Japanese ethnicity, and pre-existing ILD. The onset ranges from 40 to 53 days, but over 70% occur within the first 2 months of therapy [55]. A short interval between drug initiation and pneumonitis onset, diffuse alveolar damage pattern on computed tomography (CT) of the chest, and pre-existing ILD are associated with poor prognosis [14]. Management is based on severity, and, similar to ICIs, it involves drug cessation and observation for Grade 1, oral corticosteroids for Grade 2, and hospitalization and intravenous steroids therapy is recommended for severe disease with Grade 3 and 4 [14]. For rechallenge, an alternate TKI is recommended.

## 4. Radiation-Induced Lung Injury

Radiation to the thorax is integral to many cancer treatment regimens (both curative and palliative), and it is frequently seen with protocols for lung cancer, breast cancer, lymphoma, or total body irradiation in preparation for bone marrow transplant. Radiation-induced lung injury (RILI) represents dose-limiting toxicity for thoracic radiotherapy [56]. The incidence of RILI ranges from 5 to 20%, but the data suggests that RILI incidence may be as high as 30% in patients with lung cancer [56,57]. The evolution of spatial three-dimensional radiation planning and the advent of particle therapy has helped to focus radiation beams to the tumor itself; however, even the most advanced technologies cannot eliminate incidental exposure to healthy tissue [58]. Various dosimetry parameters can influence the occurrence of RILI, including exposure volume, mean lung dose, effective dose, treatment schedule, and tumor location [57]. RILI rarely occurs with fractionated doses below 18–20 Gy, but doses above this threshold can injure alveoli, resulting in impaired gas exchange and altered cellular function [57]. The inflammatory response triggers a cycle of increased inflammation, vascular permeability, and cytokine release, and perpetuation of a non-healing tissue response can lead to chronic radiation injury [59]. Typically, RILI occurs in doses higher than 40 Gy; however, since radiation may be part of multi-modality therapy, including immunotherapy, ADCs, and biologically targeted agents, lung injury may occur even at lower doses of radiation [58]. Other factors such as advanced age, smoking history, tumor location (central or lower lobe), pre-existing lung disease, prior thoracic irradiation, and concomitant anti-neoplastic therapy may also promote the development of RILI [28,30]. Key predictors of severe RILI include pre-existing ILD, higher lung radiation dose parameters (particularly increased mean lung dose and lung V20, the percentage of total lung volume receiving > 20 Gy of radiation), concurrent systemic therapy (especially chemotherapy or ICIs), bilateral lung irradiation, and reduced baseline pulmonary reserve (notably low diffusion capacity). Among these, pre-existing ILD is the strongest risk factor, with fatal pneumonitis rates reported as high as 18% [31].

**Figure 3:**
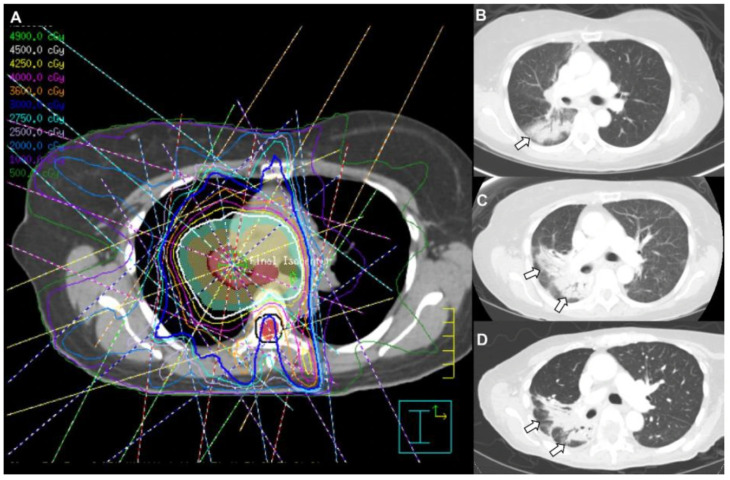
**Radiation-induced lung injury (RILI).** Radiation therapy (**A**) was delivered to the mediastinum and right upper lobe; a total of 45 Gy was delivered. The multiple colors are part of a dose distribution used in treatment planning and verfication, and warm colors (red, orange, yellow) represent higher radiation dose whereas cool colors (green, blue, purple) reflect lower radiation dose. Two months after completion of radiation treatment (**B**), new infiltrates in the right hilum and areas of ground-glass in the right lower lobe were observed. Four months after completion of radiation therapy (**C**), adenopathy had improved, but infiltrates in the paratracheal region and right lung (arrow) continued to evolve with air bronchograms and bronchiectatic changes. Six months after completion (**D**), parenchymal changes around the right hilum decreased, consistent with volume loss and traction bronchiectasis (arrows).

The development of RILI can be divided into an early state with radiation pneumonitis and an advanced stage with pulmonary fibrosis. The diagnosis of RILI can be made clinically based on the timing of symptoms, clinical history, comparative evaluation of radiation field and infiltrates, and exclusion of competing diagnoses. Acute radiation pneumonitis typically occurs as early as 6 weeks to months after exposure, and symptoms can range from mild to severe. Dyspnea and cough are common, and occasionally low-grade fever may be present. With conventional radiotherapy, estimates of acute pneumonitis for Grade 1 to 2 are 12% with Grade > 3 of <1% [58]. Early radiographic changes (Figure 3) include ground-glass opacification, diffuse haziness, or distortion of normal pulmonary markings. Other CT chest findings include conventional, mass-like consolidation with volume loss, traction bronchiectasis, and scar-like changes [56,59]. Radiation pneumonitis may rarely occur outside of the irradiated field due to radiation scatter or as a hypersensitivity response. Radiation-induced pulmonary fibrosis typically occurs more than 1 year following therapy, and estimates of pulmonary fibrosis are 10% with Grade 1 to 2 symptoms [56,58]. Radiation recall pneumonitis (RRP) is a rare, delayed radiation-induced lung toxicity, which develops within the previously irradiated field following chemotherapy (taxanes, gemcitabine, anthracyclines, alkylating agents, or pyrimidine analogs), molecular targeted therapies, and ICIs, as well as other medications (tamoxifen, simvastatin, levofloxacin, or isoniazid) [19,59]. RRP usually occurs with initiation of the offending agent but may surface after several courses of therapy. The treatment of RRP entails the discontinuation of the precipitating agent, glucocorticoids, and supportive care.

Glucocorticoids remain the cornerstone for treatment of acute RILI, and there are ongoing studies using other agents to mitigate toxicity [57,60]. Standard treatment regimen includes prednisone 40 mg/day for 2 weeks, followed by tapering over 4 to 6 weeks [60].

## 5. Pleural Disease

Pleural involvement is common in patients with cancer and may mimic both infectious and malignant disease. Parapneumonic and malignant effusions are typically exudative, and pleural fluid analysis can guide management [61,62]. Although pleural fluid cytology may be helpful, diagnostic sensitivity varies by the underlying tumor [63]. Clinical correlation is essential, and unexplained exudative effusions often warrant additional diagnostic evaluation to exclude malignancy. Moreover, multiple therapies and disease processes can cause pleural disease and may be exudative on pleural fluid analysis. 

### 5.1. Radiation-Induced Pleural Effusion

Radiation pleuritis or pleural effusion may develop weeks to months following thoracic radiation. The effusion is often unilateral and confined to the irradiated field [12,64]. Clinical presentation includes dyspnea, low-grade fever, and pleuritic chest pain, and it can overlap with possible infectious pneumonia. CT chest findings may show pleural thickening or small effusion adjacent to parenchymal changes in radiation pneumonitis or fibrosis. Radiation injury to the lymphatics, pleural surfaces, and pulmonary vasculature can lead to inflammation and fibrosis [58]. Dosimetric factors that can contribute include whole lung V5 (volume receiving > 5 Gy), combined and ipsilateral mean lung dose, V5-V50, and mean heart dose [64,65]. Most cases are self-limited; however, corticosteroids may be beneficial in symptomatic patients. In those with large recurrent pleural effusion, other interventions (indwelling pleural catheter, pleurodesis) may be indicated [62].

### 5.2. Therapy-Related Pleural Effusion

Several agents are associated with the development of pleural effusions (Table 2), including dasatinib, gemcitabine, docetaxel, methotrexate, and bleomycin [66,67]. Dasatinib-associated pleural effusions are typically exudative, lymphocyte-predominant, and may occur months after therapy initiation. These are often resolved with drug interruption, corticosteroids, or diuretics [68]. Docetaxel is associated with fluid retention and capillary leak syndrome, and corticosteroid therapy with docetaxel administration has proven to help reduce fluid retention [69].

Selective RET inhibitors, particularly selpercatinib, have been linked to the development of chylothorax and chylous ascites [70]. The effusion may appear milky and opaque, sometimes leading to misinterpretation as empyema. The diagnosis is confirmed by triglyceride levels > 110 mg/dL or detection of chylomicrons in the pleural fluid [61,62]. Drug cessation, dose reduction, or switching to an alternative agent may be beneficial.

Rarely, pleural effusions can occur with ICIs; these are typically exudative, sometimes eosinophilic, and IgG4-related pleural disease has also been reported [71,72]. It is important to differentiate effusion from malignancy and/or pseudoprogression, but these typically respond to corticosteroids and drug cessation.

## 6. Pulmonary Vascular Disease

Pulmonary hypertension (PH) may be the byproduct of various conditions, including anti-neoplastic therapies, cardiac dysfunction, acute hypoxic respiratory insufficiency, chronic thromboembolic disease, myeloproliferative neoplasms, and chronic hypoxia.

Pulmonary arterial hypertension has been reported with dasatinib and proteosome inhibitors (carfilzomib, bortezomib) [73,74,75]. Workup with right heart catheterization to confirm the diagnosis, drug cessation, and use of pulmonary vasodilator therapy is recommended.

Cardiotoxic agents, including anthracyclines and certain targeted therapies, can cause left ventricular dysfunction and heart failure, leading to PH associated with left-sided heart disease. DILI from bleomycin, busulfan, and thoracic radiation may cause acute lung injury and interstitial pulmonary fibrosis with resultant hypoxia, leading to PH associated with lung diseases and/or hypoxia. Cancer therapies associated with increased thromboembolic risk include platinum-based agents, tamoxifen, immunomodulatory agents (thalidomide, lenalidomide, and pomalidomide), EGFR-targeted antibodies (cetuximab and panitumumab), second- and third-generation BCR-ABL inhibitors, and cyclin-dependent kinase inhibitors (particularly abemaciclib), which could lead to PH associated with chronic pulmonary artery obstruction.

### Pulmonary Veno-Occlusive Disease

Pulmonary veno-occlusive disease (PVOD) is a rare cause of pulmonary hypertension characterized by fibrous obliteration of pulmonary venules and small pulmonary veins. It has been reported following exposure to several chemotherapeutic agents, including bleomycin, mitomycin C, carmustine, cisplatin, and cyclophosphamide, as well as after hematopoietic stem cell transplantation. Among these, mitomycin C is the best-established risk factor, with reported incidence rates of approximately 7–10% in selected cohorts, whereas PVOD associated with other agents has been described mainly in isolated case reports, and its true incidence remains unknown but appears to be exceedingly low. Clinically, PVOD presents insidiously with progressive dyspnea, fatigue, hypoxemia, and a markedly reduced diffusing capacity. High-resolution computed tomography typically demonstrates a triad of centrilobular ground-glass nodules, smooth interlobular septal thickening, and mediastinal lymphadenopathy; however, this pattern is non-specific and may overlap with pulmonary edema, lymphangitic carcinomatosis, or drug-induced pneumonitis. Consequently, diagnosis is usually based on a combination of clinical features, suggestive imaging, precapillary pulmonary hypertension on right heart catheterization, and the development of pulmonary edema following pulmonary vasodilator therapy, as lung biopsy carries substantial risk. Management is largely supportive, with early referral to specialized PH centers and cautious use of vasodilators, while lung transplantation remains the only definitive curative option and should be considered early, given the poor prognosis.

## 7. Other Conditions

### 7.1. Transfusion-Related Lung Injury

Transfusion-related acute lung injury (TRALI) is a serious, potentially fatal complication of blood product transfusion. Patients rapidly develop hypoxemic respiratory insufficiency, bilateral pulmonary infiltrates, and sometimes fever and chills [76]. TRALI is a clinical diagnosis with non-hydrostatic pulmonary edema and a temporal relation to the transfusion. TRALI must be differentiated from transfusion-associated circulatory overload (TACO). TACO represents hydrostatic pulmonary edema, and signs and symptoms of volume overload are often present. Risk factors include compromised cardiac function, renal dysfunction, emergent surgical intervention, and rapid blood product administration [77]. Supportive respiratory measures including supplemental oxygen, high-flow oxygen, non-invasive ventilation or even mechanical ventilation may be required.

### 7.2. Cryptogenic Organizing Pneumonia

Cryptogenic organizing pneumonia (COP), previously known as bronchiolitis obliterans with organizing pneumonia, is a noninfectious lung condition that has no identifiable cause and is classified as a form of idiopathic organizing pneumonia [78]. It is often suspected when patients with presumed infection fail to respond to antimicrobial therapy. Secondary organizing pneumonia may arise after an infection (e.g., bacterial or viral, including COVID-19), anti-neoplastic therapy (e.g., bleomycin, ICIs), hematopoietic stem cell transplantation, and radiation injury or as a reaction to other lung processes. While dry cough and dyspnea are the most common symptoms, fever, chills, fatigue, and other non-specific viral-like symptoms may also be present.

**Figure 4:**
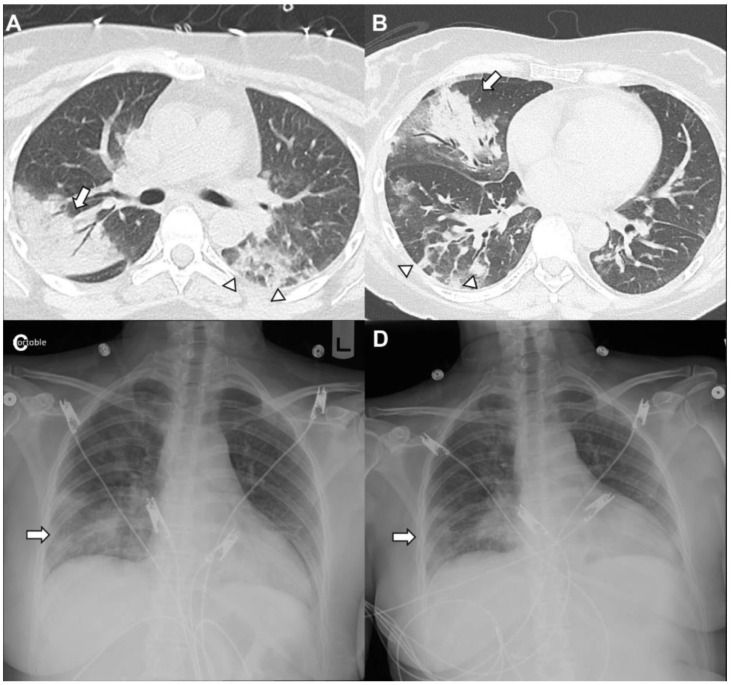
**Cryptogenic organizing pneumonia.** Lymphoma treated with obinutuzumab and lenalidomide presenting cryptogenic organizing pneumonia. Computed tomography of the chest (**A**) revealed consolidative opacities in the right upper lobe (arrow) and ground-glass (arrowhead) peripheral infiltrates on the left. She returned 2 weeks later with low-grade fever and cough; repeat imaging (**B**) showed bilateral multilobar ground-glass (arrowheads) and consolidative opacities (arrow) in the peribronchovascular and peripheral distribution (right greater than left) with a migratory pattern. Chest radiograph on readmission (**C**) showed infiltrates (arrow) in the right middle and lower lobes which improved days later (**D**, arrow) after steroid therapy.

Imaging may reveal nodular lesions (which can mimic neoplasm), ground-glass opacities, or patchy consolidations in peribronchovascular, peripheral, or band-like distributions [79]. These infiltrates may also be migratory (Figure 4), and conditions such as hypersensitivity pneumonitis, eosinophilic pneumonia, alveolar hemorrhage, and vasculitis should be excluded prior to considering COP [78]. Bronchoscopy can help rule out infection, and bronchoalveolar lavage often reveals a lymphocyte predominance. Some patients may improve spontaneously with close observation. Symptomatic patients are typically treated with corticosteroids, using a slow taper as the initial intervention.

### 7.3. Other Risk Factors

ILAs are important noninfectious pulmonary parenchymal findings on CT chest that require deliberate identification. They are defined as bilateral, non-dependent parenchymal lung abnormalities such as ground-glass opacities, reticulations, architectural distortion, traction bronchiectasis, and/or honeycombing, which do not meet criteria for ILD [80]. Approximately 46% of ILAs may progress to ILD [81]. In patients with cancer receiving immunotherapies, targeted therapies, or ADCs, ILAs have been associated with an increased risk of DILI [49].

## 8. Conclusions

Pulmonary complications of cancer and its therapies encompass a broad spectrum of diseases. A systematic approach, with judicious application of diagnostic and invasive testing, should be applied. A comprehensive understanding of their clinical presentations, radiographic findings, and natural history will guide both accurate diagnosis and effective management.

## Figures and Tables

**Table 1:** Grading system for lung toxicity [1,2].

Grade	CTCAE v6.0	RTOG
0	No changes	No changes
1	Asymptomatic, only radiographic findings	Asymptomatic or mild symptoms
2	Symptomatic, does not interfere with daily activities	Moderate symptoms of pneumonitis (severe cough) and radiographic changes
3	Symptomatic, interferes with daily activities, requires supplemental oxygen	Severe symptoms of pneumonitis, dense radiographic changes
4	Threatens life, requires mechanical ventilation support	Symptoms of severe respiratory failure requiring assisted ventilation or continuous oxygen
5	Death	Death-related late effects of radiotherapy

CTCAE v 6.0, Common Terminology Criteria for Adverse Events version 6.0; RTOG, Radiation Therapy Oncology Group.

**Table 2:** Patterns of drug-induced lung injury and associated agents.

Pulmonary Manifestation	Chemotherapy	Molecular
Bronchospasm	Alkylating agents (cyclophosphamide), antitumor antibiotics, anti-metabolities, taxanes, ATRA	Monoclonal antibodies
DAD, NCPE, DAH	Alkylating agents, antitumor antibiotics, anti-metabolites, taxanes, platinoids (oxaliplatin), ATRA	Immunomodulators, monoclonal antibodies, proteosome inhibitors, rapamycin inhibitors, tyrosine kinase inhibitors
Eosinophilic pneumonia	Alkylating agents, antitumor antibiotics, anti-metabolites, taxanes	Monoclonal antibodies (rituximab)
Hemoptysis		Immunomodulators, monoclonal antibodies, proteosome inhibitors
Interstitial pneumonitis/fibrosis	Alkylating agents, antitumor antibiotics, anti-metabolites, taxanes, platinoids (oxaliplatin)	Immunomodulators, proteosome inhibitors, rapamycin inhibitors, tyrosine kinase inhibitors, antibody–drug conjugates, CDK inhibitor, PI3K, PARP inhibitor
Pleural effusion	Alkylating agents (cyclophosphamide), anti-metabolites (methotrexate, gemcitabine), taxanes, ATRA	Tyrosine kinase inhibitors
Pulmonary hypertension	Antitumor antibiotics (bleomycin, mitomycin)	Proteosome inhibitors, tyrosine kinase inhibitors
Radiation recall	Alkylating agents (nitrosoureas), anti-metabolites, taxanes,	Monoclonal antibodies, proteosome inhibitors, tyrosine kinase inhibitors (gefitinib, EGFR)

DAD, diffuse alveolar damage; NCPE, noncardiogenic pulmonary edema; DAH, diffuse alveolar hemorrhage; EGFR, epidermal growth factor receptors; ATRA, all-trans retinoic acid; CDK, cyclin-dependent kinase; PI3K, phosphoinositide 3-kinase.

## Data Availability

No new data were created or analyzed in this study. Data sharing is not applicable to this article.

## Abbreviations

The following abbreviations are used in this manuscript:

DILI	drug-induced lung injury
ICI	immune checkpoint inhibitor
ARDS	acute respiratory distress syndrome
VEGF	vascular endothelial growth factor
EGFR	epidermal growth factor receptor
irAE	immune-related adverse event
ADC	antibody–drug conjugate
RILI	radiation-induced lung injury
ILD	interstitial lung disease
RRP	radiation recall pneumonitis
CT	computed tomography
PH	pulmonary hypertension
PVOD	pulmonary veno-occlusive disease
TRALI	transfusion-related acute lung injury
TACO	transfusion-associated circulatory overload
COP	cryptogenic organizing pneumonia
PAP	pulmonary alveolar proteinosis
GM-CSF	granulocyte-macrophage colony-stimulating factor
ILA	interstitial lung abnormalities
CTCAE	Common Terminology Criteria for Adverse Events
RTOG	Radiation Therapy Oncology Group
BCNU	carmustine
CCNU	lomustine
PD-1	programmed cell death protein 1
PD-L1	programmed death-ligand 1
CTLA-4	cytotoxic T-lymphocyte-associated protein 4
ALK	anaplastic lymphoma kinase
TKI	tyrosine kinase inhibitors
